# Total body irradiation dose optimization based on radiological depth

**DOI:** 10.1120/jacmp.v13i3.3767

**Published:** 2012-05-10

**Authors:** Amjad Hussain, Peter Dunscombe, J. Eduardo Villarreal‐Barajas, Derek Brown

**Affiliations:** ^1^ Department of Medical Physics, Tom Baker Cancer Centre University of Calgary; ^2^ Department of Radiation Oncology, Tom Baker Cancer Centre University of Calgary; ^3^ Department of Physics and Astronomy University of Calgary Calgary AB Canada

**Keywords:** TBI, DMLC, radiological depth, translating bed, dose optimization

## Abstract

We have previously demonstrated the use of Eclipse fluence optimization to define aperture sizes for a novel aperture modulated translating bed total body irradiation (TBI) technique. The purposes of the present study were to identify, characterize, and correct for sources of error inherent in our previous fluence optimization technique, and to develop a clinically viable fluence optimization module for the translating bed TBI technique. Aperture modulated TBI is delivered by translating the patient at constant speed on a custom bed under a modulated radiation beam. The patient is then turned from supine to prone and the process repeated, resulting in an AP‐PA treatment. Radiological depths were calculated along divergent ray lines through individual CT slices of a RANDO phantom. Beam apertures, defined using a dynamic multileaf collimator (DMLC), were generated using calculated radiological depths and calibration factors that relate fluence to aperture size in a dynamic environment. These apertures were defined every 9 mm along the phantom superior‐inferior axis. The calculated beam apertures were further modified to account for scatter within the patient. For dose calculation purposes the individual MLC files were imported into Eclipse. For treatment delivery, dynamic MLC files for both AP and PA beams were generated and delivered dynamically. Dose homogeneity in the head and neck region of the RANDO phantom was within ± 4% of the prescribed dose with this novel technique compared to −5% to +7% with our previous aperture modulated technique based on Eclipse fluence optimization. Fluence optimization and beam aperture calculation using the new technique offers a ten‐fold reduction in planning time and significantly reduces the likelihood of user error during the planning process. In conclusion, a clinically viable aperture modulated translating bed TBI technique that employs dynamically shaped MLC‐defined beam apertures based on radiological depth calculations, has been developed.

PACS numbers: 87.55.‐x, 87.55.D‐

## I. INTRODUCTION

Total body irradiation (TBI) has been used for many years, both as a conditioning regimen for bone marrow transplantation (BMT) and for eradicating malignant cells.^(^ ^1^ ^–^ ^4^ ^)^

An established requirement in TBI is to deliver a uniform dose to the entire patient body. Unlike standard radiotherapy techniques, achieving dose uniformity during TBI is a challenging task. Internal inhomogeneities, irregular body contours, and the large target size often result in dose uniformity no better than ±10%, a dose criterion that has been considered acceptable for TBI regimens.^(^ ^4^ ^–^ ^6^ ^)^ Nonuniform dose distributions can compromise the clinical outcome either through a lower dose to the patient or higher doses to critical organs (e.g., lungs, kidneys). A number of linac‐based TBI techniques have been developed to improve dose uniformity. One such method is to compensate for external contour variation using bolus around the patient or using copper blocks of variable thicknesses mounted to the head of the linac.^(^ ^4^ ^,^ ^7^ ^,^ ^8^ ^)^ Some investigators have used compensation based on water columns of different heights.^(^ ^9^ ^,^ ^10^ ^)^ Physical compensators, however, are labor intensive to fabricate, and bolus may be uncomfortable for the patient and difficult to reproduce. Chrétien et al.^(^ ^11^ ^)^ developed a unique translating bed technique that uses variable velocity to improve dose uniformity along the superior–inferior (sup–inf) axis of the patient. Despite large improvement in dose uniformity along the sup–inf axis, the variable velocity technique with rectangular fields cannot be used to improve dose uniformity in the transverse plane of the patient. More recently, helical tomotherapy has been used for the application of techniques such as intensity‐modulated total marrow irradiation (IM‐TMI) and total lymphatic irradiation for escalating dose to the bones and lymphatic system.^(^ ^3^ ^,^ ^12^ ^,^ ^13^ ^)^

Recently, we described a novel aperture modulated TBI (AM‐TBI) technique^(^ ^14^ ^,^ ^15^ ^)^ for application with a linear accelerator. During AM‐TBI, a dynamic multileaf collimator (DMLC) varies the two‐dimensional beam aperture while the patient is translated on a moving bed under a vertical radiation beam. Despite improved dose uniformity, the technique is not without shortcomings, with several issues limiting the accuracy of the fluence optimization, as well as impeding clinical deployment of the technique. While these issues are discussed in detail elsewhere,^(^ ^15^ ^)^ a brief description of some of the challenges with AM‐TBI is provided, as follows:

1. The Eclipse‐based technique requires calculation of optimized fluence distribution using multiple static beams, making it difficult to implement clinically.
2. The beam divergence effects resulting from the use of multiple static beams causes deviations from the optimal fluence along the sup–inf axis of the RANDO phantom.
3. Errors in fluence optimization also result from the nonuniform beam profile in the sup–inf direction.

In the current work, an advanced aperture modulated translating bed TBI technique has been developed. Radiological depths are calculated on individual RANDO phantom CT slices in MATLAB. Based on these radiological depths, beam apertures are designed using the DMLC. This novel TBI technique is referred to as aperture modulation based on Radiological Depths (RD‐TBI) and is compared throughout the paper to our previous technique, based on Eclipse fluence optimization, referred to as AM‐TBI.

## II. MATERIALS AND METHODS

**Figure 1:**
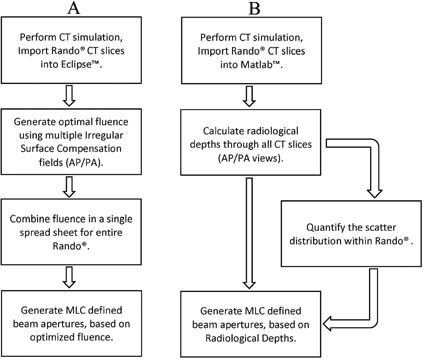
Flowcharts summarizing the (A) fluence calculation and aperture modulation for AM‐TBI and (B) radiological depth calculation and aperture modulation for RD‐TBI.

A flowchart summary of the both AM‐TBI and RD‐TBI techniques is given in Figs. 1(A) and (B), respectively.

### A. CT simulation and contouring

**Figure 2:**
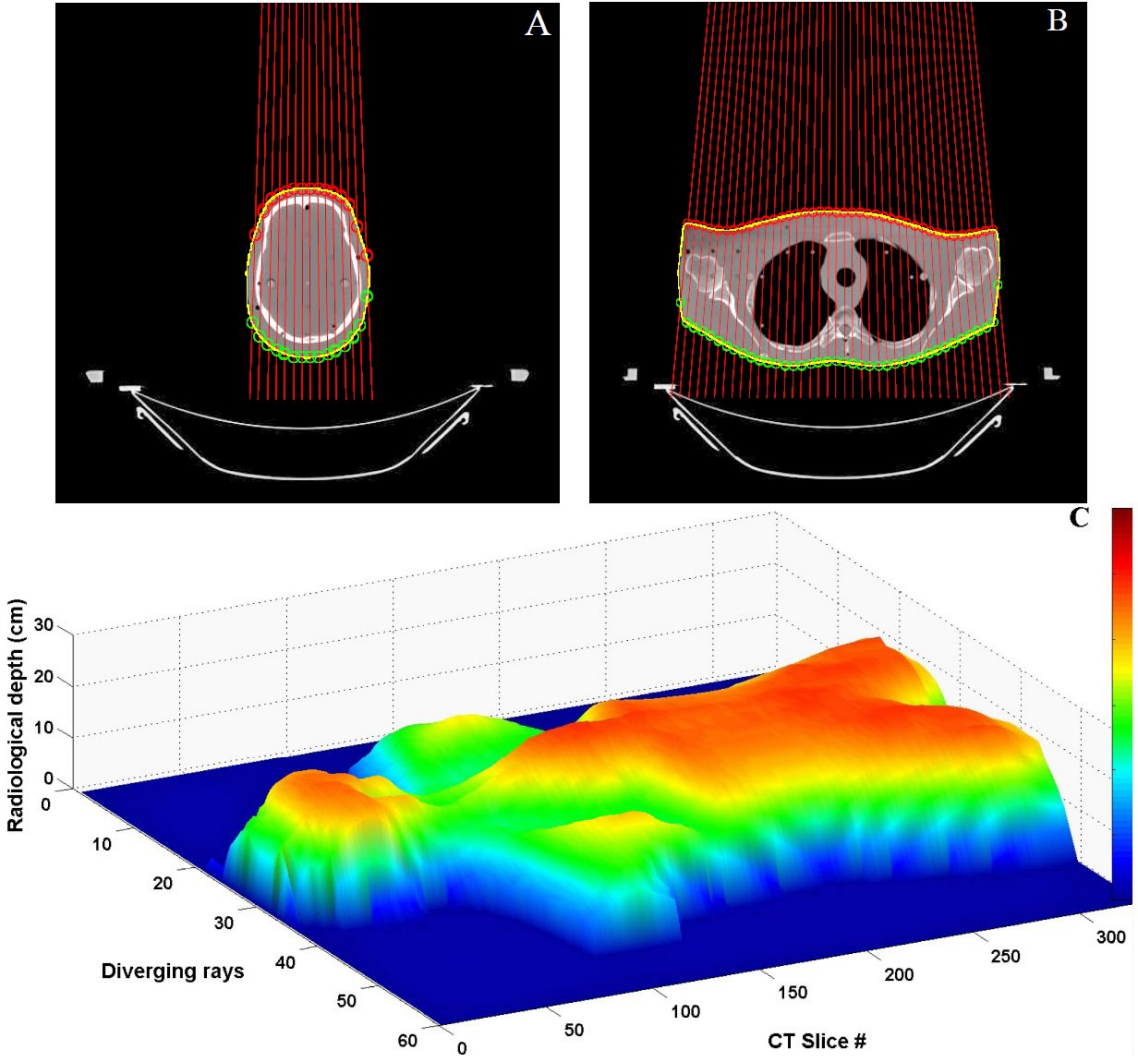
Diverging rays through CT slices at (A) head and (B) lung position. The contours are represented by yellow lines. The red and green circles indicate the entrance and exit points, respectively. Radiological depth map (C) for the entire RANDO, AP view only.

An anthropomorphic RANDO phantom (The Phantom Laboratory, Salem, NY) was CT scanned using a Brilliance Big Bore spiral CT simulator (Philips, Inc.) with 3 mm slice thickness. All CT slices were imported into MATLAB (The MathWorks, Inc., Natick, MA) and an external body contour was defined. From the body contour, entrance and exit points were determined for diverging rays, as shown in Fig. 2.

### B. Radiological depth determination

The diverging rays start at the X‐ray source position and pass through the plane of a CT slice. The distance separating these ray lines in the transverse plane at isocentre (100 cm) is equal to the MLC leaf width. Figures 2(A) and (B) illustrate the diverging rays passing through CT slices at the head and lung positions, respectively. Entrance and exit points for each ray passing through the contour are determined.

The radiological depth along each ray line, *d*, is calculated using the following well‐known equation:

(1) $$d\prime =\frac{\sum_{i=1}^{n}d_{i}\rho_{i}}{\rho_{water}}$$

where $d_{i}$ is the length of a segment with electron density $\rho_{i}$ along the ray line through the CT slice. *d′* is calculated for every ray line and over all CT slices.

The final radiological depth map for the entire RANDO phantom is presented in Fig. 2(C). The depths towards the body edges and through the lungs are shorter than the rest of the body, as expected. Radiological depth is calculated on a slice by slice basis so the radiological depth, and hence the fluence optimization, are not degraded by beam divergence in the sup–inf direction.

### C. Aperture modulation

**Figure 3:**
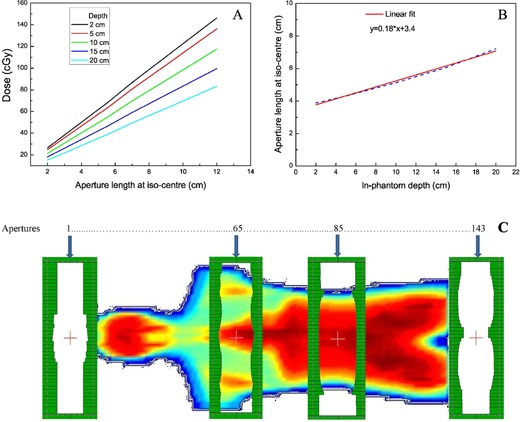
Cumulative dose (A) measured in water phantom against increasing aperture length at different depths; aperture length vs. depth (B) to deliver a 100 cGy; apertures distribution (C) as seen through beam's eye view for dose calculation and dose delivery during RD‐TBI. The numbers on top indicate selected apertures and their locations.

Because the patient is translated at a constant speed, a larger aperture in the sup–inf direction means that a point in the patient/phantom is in the beam for a longer period of time. Dose homogeneity is achieved by matching radiological depth to points in the plane of interest to aperture length — longer sup–inf apertures for thicker sections and vice versa. The aperture is then shaped dynamically as the patient is translated through the beam. To deliver a uniform dose, one requires knowledge of the relation between aperture length along the sup–inf direction and different phantom depths, for constant dose. To determine this relationship, cumulative absolute doses were measured (Fig. 3(A)) at different depths for various aperture lengths in a solid water phantom with an Exradin A12 ionization chamber. The solid water phantom was placed on a bed moving at 15 cm/min under a vertical radiation beam with 300 MU/min dose rate. There is a linear relation between aperture length and absorbed dose for a given radiological depth. For the same aperture length along the direction of motion of phantom, the absorbed dose decreases with increasing depth. Combining these two concepts, aperture length can be adjusted to deliver similar doses at different depths in the phantom. Such a relation (given in Fig. 3(B) and Eq. (2)) is derived from Fig. 3(A) data to deliver 100 cGy to different depths from two opposed beams.

(2) MLC leaf separation =0.18×Radiological Depth+3.4

Leakage through MLCs was not evaluated explicitly but was implicitly determined while generating Eq. (2). Beam apertures can then be designed along the sup–inf axis of the RANDO phantom at 9 mm intervals. To cover the 99 cm long RANDO phantom, 143 (17 + 109 + 17) MLC defined apertures were designed for each AP and PA irradiation. The number 143 corresponds to the length of the phantom (109 × 0.9 = 99 cm) plus 15 cm (17 × 0.9) on both ends for complete irradiation coverage. Selected apertures along RANDO are shown in Fig. 3(C). The numbers on the top of the figure indicate the sequence of these apertures.

### D. Scatter contribution

To account for the scatter contribution, scatter kernels, *K* were derived using the concept of scatter maximum ratios (*SMR*).^(^ ^4^ ^)^ The mathematical expression is given in Eq. (3) below:

(3) $$SMR(d,\;r_{d})=TMR(d,\;r_{d})\left(\frac{S_{p}(r_{d})}{S_{p}(0)}\right)-TMR(d,0)$$

where $r_{d}$ is the field size at depth *d*, $S_{p}$ is the phantom scatter factor, and *TMR* is the tissue maximum ratio. The scatter kernel *K* is determined using the following equation:

(4) $$K=SMR(d_{ref},r_{2})-SMR(d_{ref},r_{1})$$

where $d_{ref}$ is set at 10 cm, and $r_{2}$ and $r_{1}$ are the radii of equivalent circular fields related to rectangular fields with length *X* and width *Y* as follows:

(5) $$\begin{array}{c} r_{2}=\frac{1.128.X.(Y+\frac{1}{2})}{X+Y+\frac{1}{2}}\\ r_{1}=\frac{1.128.X.(Y-\frac{1}{2})}{X+Y-\frac{1}{2}} \end{array}$$

The radii are calculated from square beam data with the assumption that the area of both the equivalent circle and equivalent square are the same.^(^ ^4^ ^)^

**Figure 4:**
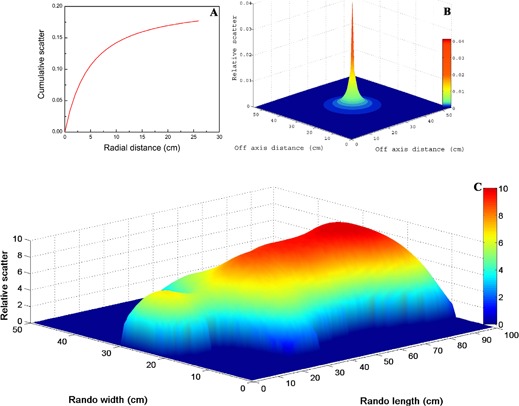
Cumulative SMR (A) as a function of the beam radius; schematics (B) of the scatter kernel at 10 cm depth against radius of the beam; elative scatter distribution (C) in RANDO phantom, moving under a vertical radiation beam.

Figures 4(A) and (B) show the cumulative *SMR* and shape of the scatter kernel at 10 cm depth vs. radius of the beam, respectively. It is clear from Eqs. (4) and (5) that the shape of the kernel is a function of field size. During the current TBI procedure the field width is kept constant, whereas the length varies by approximately 10%. To a good approximation for the purposes of this calculation it may be assumed that, on average, the equivalent field area is constant.

In order to determine the scatter distribution within the RANDO phantom, a convolution of the scatter kernel, *K*, with the radiological depth RD is performed using Eq. (6):

(6) $$Relative\;Scatter(x,\;y)=\iint_{\left(u;v)\in (RD\right)}RD(u,\;v)K(u-x,\;v-y)dudv$$

The resultant scatter distribution is shown in Fig. 4(C).

In order to include the scatter contribution in the fluence optimization, all the beam apertures were scaled using the following equation:

(7) $$Optimized\;MLC\;leaf\;separation=\frac{MLC\;leaf\;separation}{1-\left(\frac{S_{\max}-S}{100}\right)}$$

where *MLC* leaf separation is the one calculated using Eq. (2), $S_{\max}$ corresponds to the maximum relative scatter, and *S* is the relative scatter at any point in RANDO, as shown in Fig. 4(C). The maximum scatter, as expected, was observed in the abdomen.

### E. Dose distribution calculation

Due to the limitations of the treatment planning system for dose calculations in a dynamic environment, the dynamic irradiation was simulated as a number of static beam positions, as shown in Fig. 3(C). The beams were defined every 9 mm along RANDO to deliver a 100 cGy dose to the midplane. For dose distribution calculations, individual MLC files for all AP/PA beams were imported into Eclipse treatment planning system. Each aperture was calculated with 18 monitor units (MU), mimicking a constant dose rate (300 MU/min) and constant bed velocity (15 cm/min).

Deviation of the calculated dose from the prescription (100 cGy) was determined using the following equation:

(8) $$Deviation\;(\%)=\frac{\left(D_{calculated}-D_{prescribed}\right)}{D_{prescribed}}\times 100$$

### F. Translating bed and dynamic TBI delivery

A novel translating bed for TBI has been designed in‐house^(^ ^14^ ^)^ with positional accuracy of ±0.1 mm. The speed and precise position of the bed are monitored in real time by a dual encoder system during irradiation. The radiation beam and bed motion are synchronized, such that if one fails, the other shuts off or stops immediately. The distance from the source to the bed surface is 205 cm. With bed motion in the cross‐plane direction, a collimator rotation of 90° was necessary to accomplish aperture modulation in the sup–inf direction. The *Y* jaws were positioned to cover entire width of the RANDO phantom in the lateral direction.

During irradiation the apertures are dynamically modified as the phantom/patient is translated through the beam. Multiple static, MLC defined apertures are combined into a single dynamic MLC file for dynamic dose delivery. Every control point of the dynamic MLC file represents a single beam aperture, and because of the constant bed velocity and dose rate, each control point is equally weighted. Two such files were created, one for each AP and PA dose delivery. For a given dose rate and bed velocity, the treatment time was calculated in terms of MU. Clearly setup uncertainties and positioning uncertainties could compromise the uniformity of the dose distribution within the phantom/patient. This issue has been discussed elsewhere and will not be further addressed here.^(^ ^15^ ^)^

## III. RESULTS

### A. Dose calculation using RD‐TBI technique without scatter correction

Treatment plans were created by importing 143 AP/PA individual MLC files into Eclipse to deliver a 100 cGy dose to the midplane of RANDO. Each aperture was delivered with the same number of monitor units (18 MU each). For each AP/PA position, a total of 2574 MU was delivered within a total “treatment” time of 17 minutes. The treatment time for this technique is twice that of our standard TBI technique. However, using larger width apertures, the treatment time can be reduced at the expense of decreased dose uniformity.

**Figure 5:**
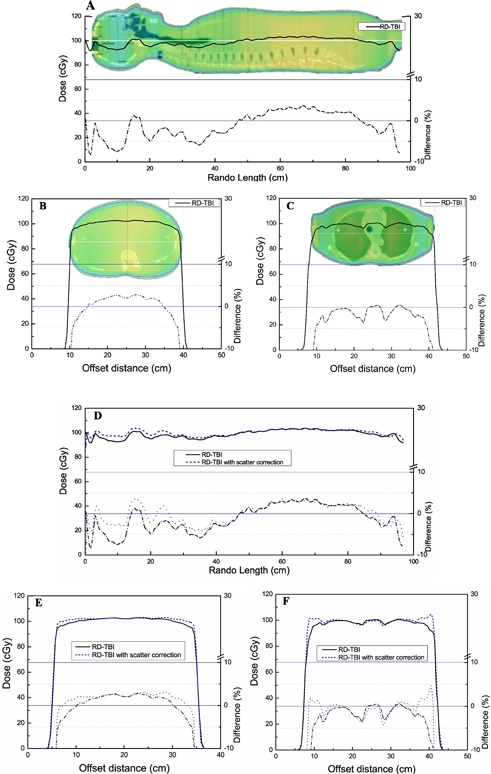
Dose profiles calculated in Eclipse, using RD‐TBI technique without scatter correction: along sup–inf axis (A), through abdomen (B), and through lungs (C); a dosimetric comparison ((D), (E), and (F)) between RD‐TBI techniques with and without scatter correction along sup–inf axis through abdomen, and through lungs, respectively. The dose deviation from the prescription (100 cGy) is plotted against right axis.

Figure 5(A) shows the dose profile calculated in Eclipse along RANDO at midplane, using MLC sequences modulated with the RD‐TBI technique with no scatter correction. The dose deviation from the prescription (100 cGy) is plotted as a dotted line against the right axis, calculated using Eq. (8). The calculated dose varies from −7% to +3% along the suf‐inf axis of RANDO.

Figures 5(B) and (C) show the representative dose profiles through the abdomen and lung transverse sections in RANDO at midplane. Dose towards the body edges is lower compared to the central plane.

### B. Dose calculation using RD‐TBI technique with scatter correction

The calculated beam apertures were then modified to account for the scatter contribution using Eq. (7). These optimized beam apertures were imported into Eclipse for dose calculations in RANDO. The dose distribution calculation performed using the RD‐TBI technique, corrected for scatter, is shown in Figs. 5(D), (E), and (F). In order to demonstrate improvement in the dose homogeneity, dose profiles are compared against RD‐TBI without scatter correction. The dose deviation from the prescription is plotted against the right axis, calculated with Eq. (8). Figure 5(D) illustrates calculated sup–inf dose profiles along RANDO at midplane. Dose deviation from the prescription is less than ±4% for the RD‐TBI with scatter correction. Figures 5(E) and (F) show a comparison of dose profiles through the abdomen and lung sections, respectively. Dose uniformity through the abdomen and lungs is approximately ±2% when the scatter correction is applied.

### C. Dosimetric comparison to AM‐TBI

The doses calculated by RD‐TBI with scatter correction applied were compared against those calculated by AM‐TBI (see Hussain et al.^(^ ^15^ ^)^ for details).

**Figure 6:**
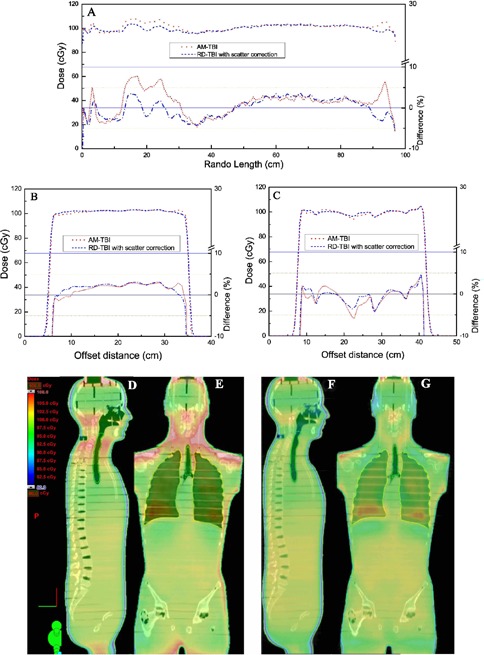
Dosimetric comparison between AM‐TBI (red line) and RD‐TBI optimized for scatter (blue line): along sup–inf axis (A), through abdomen (B), and through lungs (C). Sagittal and coronal planer dose distributions in RANDO calculated with AM‐TBI ((D) and (E)), and calculated with RD‐TBI corrected for scatter ((F) and (G)).

A dose distribution comparison was performed in three dimensions in RANDO. Figures 6(A), (B), and (C) illustrate improved dose homogeneity in the calculated dose distribution using RD‐TBI technique corrected for scatter compared to the previous AM‐TBI technique.^(^ ^15^ ^)^ Calculated dose uniformity in the head and neck region of the RANDO phantom was within ±4% of the prescribed dose, with RD‐TBI technique corrected for scatter compared to −5% to +7% with AM‐TBI. These latter results are consistent with those previously reported by us^(^ ^15^ ^)^ to within the reproducibility of the user defined profile location.

Figures 6(D) and (E) visually demonstrate the absolute dose distributions in the sagittal and coronal planes, respectively, for AM‐TBI. Figures 6(F) and (G) show dose distributions in the same planes for RD‐TBI with scatter correction. Dose in the neck and shoulder region with RD‐TBI was, on average, 5% higher than the prescription compared to 10% with AM‐TBI.

The sagittal profiles drawn along RANDO and through the lungs in Figs. 5(A) and (C) were analyzed quantitatively. Table 1 provides values for the standard and mean deviation of the calculated dose from the prescribed dose (100 cGy). Δ is calculated using the following equation, from Venselaar et al.:^(^ ^16^ ^)^

(9) Δ=1.5×SD+|Mean|

**Table 1:** Quantitative analysis of the dose profiles plotted along RANDO and through lungs in Figs. 5(A) and (C).

*Technique*	*Mean (%)*	*Sup–Inf SD (%)*	Δ(%)	*Mean (%)*	*Lungs SD (%)*	Δ(%)
AM‐TBI	0.9	2.9	5.3	0.4	1.7	2.9
RD‐TBI^a^	0.4	2.5	4.2	0.3	1.4	2.4

a RD‐TBI technique with scatter correction

Overall RD‐TBI technique with scatter correction provides more uniform dose distributions than AM‐TBI.

### D. Improvements over AM‐TBI

With the RD‐TBI technique, instead of calculating the optimal fluence from Eclipse, beam apertures are modulated based on radiological depths and scatter corrections calculated on a slice by slice basis for the entire patient/phantom using MATLAB. Because we can automate the process in MATLAB, we significantly reduce user errors and greatly increase the efficiency of the process; the RD‐TBI reduces the amount of time spent on aperture shape design by a factor of 10. On average about 15 minutes are required to perform beam aperture optimization with this technique.

Another significant issue with AM‐TBI results from the use of fewer, larger fields for fluence optimization. This is necessary from a practical standpoint because automation of such processes is not possible in Eclipse. The use of fewer, larger fields in the AM‐TBI technique causes deviations from the optimal fluence due to beam divergence effects and the nonuniform beam profile in the sup–inf direction. These two effects, both skewing optimal fluence along the RANDO phantom, are significantly reduced in the RD‐TBI technique because radiological depths are calculated for individual CT slices. The beam aperture for the same slice is optimized based on these radiological depths, mitigating the effects of beam divergence and beam profile in the sup–inf direction.

## IV. DISCUSSION

Our results demonstrate that RD‐TBI technique, corrected for scatter, delivers a more homogeneous dose distribution to a nonuniform patient compared to AM‐TBI. The RD‐TBI technique is more practical and clinically viable. Beam aperture design with this novel technique offers a ten‐fold reduction in planning time compared to the AM‐TBI technique. No physical compensation is required, which makes it easy for the staff and comfortable for the patient.

It is probably impossible to answer the question, Do more homogeneous dose distributions in TBI result in better clinical outcomes? As with IMRT and other advanced forms of dose delivery, we presume that a more uniform distribution around the prescription point results in better outcomes. Thus it is generally assumed that every effort should be made to deliver as uniform a dose distribution as possible for TBI.^(^ ^5^ ^,^ ^6^ ^,^ ^7^ ^,^ ^8^ ^,^ ^10^ ^,^ ^11^ ^)^ This assumption was the motivation for the present study.

The dose distribution calculated by Eclipse has been validated for the AM‐TBI technique in RANDO.^(^ ^15^ ^)^ Point dose measurements were performed using LiF thermoluminescence dosimeters (TLD), placed at 29 different locations within the RANDO phantom. The phantom was then irradiated using the aperture modulated TBI technique. TLD measurements were compared with the calculations performed by Eclipse. Agreement between measurement and calculation was better than ±3%.

Figure 5(A) illustrates that dose homogeneity is approximately ±5% along the RANDO sup–inf axis. A lower dose is calculated in the head region due to the lower scatter contribution compared to the abdomen. This reduced scatter is not accounted for during the simpler aperture modulation using Eq. (2). Dose profiles through the abdomen and lungs (Figs. 5(B) and (C)) also show lower dose towards the body edges due to less scatter.

To improve the dose uniformity in three‐dimension, the scatter contribution was taken into account. Scatter kernels, *K*, were derived from the scatter maximum ratios (SMR) at 10 cm reference depth. Obviously the scattering pattern is different at different depths^(^ ^17^ ^)^ in a water phantom; however, the kernels determined at 10 cm depth are reasonable approximations of all depths and have previously been used by some investigators.^(^ ^18^ ^)^ As explained in the Methods Section, beam apertures are modified using the scatter distribution in RANDO.

Figures 5(D), (E), and (F) show that the agreement between the prescribed and calculated dose is better when the scatter correction is applied to the aperture modulation. Improvement in the dose distribution is also observed along horizontal profiles through the abdomen and lungs. Dose towards body edges is increased because the lower scatter is accounted for during aperture design.

An inherent advantage of the aperture modulated TBI is achieving dose homogeneity in the lungs without any physical shielding (Fig. 5(F)). Furthermore, with the RD‐TBI technique lungs can be partially shielded to any desired dose limit.

For this phantom study, a machine dose rate of 300 MU/min was employed for reasons of experimental efficiency. This machine dose rate results in a dose rate at midplane in the RANDO phantom of 64 cGy/min. The optimum dose rate for clinical TBI is still a matter of debate, although some experts suggest it should be in the region of 10 cGy/min. Achieving such a dose rate for clinical applications is simply accomplished by reducing the machine dose rate to the desired level while, at the same time, reducing in the same proportion the bed velocity.

A dosimetric comparison of the RD‐TBI corrected for scatter is made with AM‐TBI. Dose uniformity along RANDO midline is better with the RD‐TBI technique corrected for scatter than the AM‐TBI. Figures 6(D), (E), (F), and (G) fully demonstrate dose distributions in color shade in three orthogonal planes. There is a substantial improvement in the dose distribution toward head and neck regions with the RD‐TBI technique corrected for scatter.

## V. CONCLUSIONS

A novel, aperture modulated TBI technique that employs dynamically shaped MLC‐defined beams based on radiological depth calculation has been developed. The new TBI technique minimizes the chances of error because the radiological depth calculations are automated for all patient/phantom CT slices. Dose uniformity in RANDO is greatly improved in three dimensions without using physical compensators. Results have shown that the dose distribution achieved with the RD‐TBI corrected for scatter is better than the AM‐TBI in three‐dimension. This technique not only generates a more uniform dose distribution, but it is also faster and more clinically viable than AM‐TBI.

## ACKNOWLEDGMENTS

The authors would like to thank Marie‐Claude Lavallée, Mario Chrétien, and Luc Beaulieu of the CHUQ in Québec City, Québec, Canada for extremely useful discussions and for many helpful suggestions regarding the mechanical and electrical design of the translating bed. The authors also appreciate the thorough evaluation and thoughtful comments of the reviewers of this manuscript.
